# Disparities among smokers during the COVID-19 pandemic: Examination of COVID-19-related worries by sociodemographic factors in a U.S. Nationally representative survey

**DOI:** 10.1016/j.pmedr.2022.101835

**Published:** 2022-05-19

**Authors:** Robert T. Fairman, Scott R. Weaver, Amy L. Nyman, Lucy Popova, Zachary Massey, Reed M. Reynolds, Claire A. Spears

**Affiliations:** a School of Public Health, Georgia State University, Atlanta, GA, USA; b Missouri School of Journalism, University of Missouri, Columbia, MO, USA

**Keywords:** Health disparities, Tobacco use, COVID-19, Mental health

## Abstract

Low-socioeconomic status (SES) and certain racial/ethnic minority groups disproportionately experience tobacco-related disease and death. Underserved populations of smokers may be at disproportionate risk for elevated stress and worry related to basic needs and healthcare during the pandemic, which could impede smoking cessation and exacerbate health disparities. This study examined whether experiences with stress and worry among smokers during the COVID-19 pandemic differed by sociodemographic factors, and whether these factors predicted serious psychological distress (SPD). Data came from an October-November 2020 U.S. national representative survey of 1,223 current cigarette smokers. Analyses examined associations between sociodemographic factors with COVID-19-related worries and past-month SPD. Worry in most domains (e.g., food, housing, finances, healthcare) was more prevalent among participants with less than high school education, income less than $30,000, and those who were unemployed. Women and participants aged 30–44, with income less than $30,000, with less than high school education, not working/disabled, or on Medicaid were more likely to experience SPD. Examined separately, each COVID-19 worry predicted higher likelihood of SPD. In adjusted models, COVID-19 worries about finances (aOR = 2.3) and isolation/loneliness (aOR = 3.0) uniquely predicted SPD. Among U.S. adult smokers during the COVID-19 pandemic, those with lower SES indicated disproportionately high worry about access to basic needs and were more likely to experience SPD. Policies and interventions that address basic needs and mental health among marginalized populations of tobacco users are needed.

## 1 Introduction

Low-socioeconomic status (SES) and certain racial/ethnic minority groups experience severe health disparities, which are linked to high stress, low healthcare access, and other factors. (Myers, 2009) These populations disproportionately experience tobacco-related disease and death, with high stress contributing to smoking and lower quitting success. (U.S. National Cancer Institute, 2017) Stress is a common trigger for smoking (Perkins and Grobe, 1992, McEwen et al., 2008) and barrier to quitting, particularly among socioeconomically disadvantaged adults. (Businelle et al., 2010, Hiscock et al., 2012) The COVID-19 pandemic has disproportionately affected underserved populations; higher risk of COVID-19 infection has been associated with being Black, Hispanic/Latinx, and having housing insecurity. (Rozenfeld et al., 2020) Moreover, based on the U.S. Household Pulse Survey during the pandemic (September-December 2020), Black non-Hispanic and Hispanic adults and lower-income households were disproportionately likely to experience financial hardship. (Kim, 2021) Accordingly, underserved populations may be at particular risk for elevated stress during the pandemic, including worries about finances, housing, and healthcare. Simultaneously, the U.S. experienced heightened awareness of systemic racism and police injustice in 2020, (Dunivin et al., 2022) which could amplify race-related stress.

Research is needed to understand whether experiences with stressors in the context of COVID-19 vary by sociodemographic characteristics. For example, a U.S. nationally representative survey found that during the pandemic (April-May 2020), Hispanic adults reported greater stress about food and housing insecurity compared to whites. (McKnight-Eily et al., 2021) However, the extent to which these stressors might vary by sociodemographic characteristics specifically among smokers is unknown. This is critical because smoking cigarettes increases risk for COVID-19 progression. (U.S. National Cancer Institute, 2017, Patanavanich and Glantz, 2020) Moreover, stress and worry about COVID-19 have been commonly cited reasons for smoking and difficulty quitting during the pandemic. (Shepherd et al., 2021, Popova et al., 2021) Using a nationally representative survey of U.S. cigarette smokers, this study examined whether experiences with stress and worry during the pandemic differed by sociodemographic factors.

## 2 Methods

### 2.1 Study sample and procedures

Data come from a 2020 (October-November) survey of a national probability sample drawn from Ipsos Public Affairs’ KnowledgePanel, a probability-based web panel designed to be representative of non-institutionalized U.S. adults. Originally, KnowledgePanel used Random Digit Dialing to recruit participants, but switched to Address-Based Sampling in 2009. Panel members are provided incentives for participation in the panel and can enter raffles for prizes. (Ipsos, 2022) Adult panelists (18 + years) who reported current cigarette smoking or current electronic nicotine delivery system (ENDS) use on recent Ipsos profile surveys were randomly sampled and invited to participate upon confirmation that they were current users of cigarettes (defined as having smoked at least 100 cigarettes in their lifetime and now smoking “every day” or “some days”) or ENDS (defined as now using ENDS “every day” or “some days”), or had recently (since February 2020) quit smoking cigarettes or using ENDS. Overall, 2,752 KnowledgePanel members were invited to participate, and 1,526 completed the survey. Those who did not currently smoke cigarettes were excluded from the present analyses, resulting in a final analytic sample of 1,223 current cigarette smokers. A study-specific post-stratification weight was computed using an iterative proportional fitting (raking) procedure using benchmarks from 2019 National Health Interview Survey (gender, race/ethnicity, census region, metropolitan status, education) and KnowledgePanel profile data (household income) to adjust for sources of sampling and non-sampling error, such as panel recruitment non-response, panel attrition, and non-response to the invite to participate in this study. This study was deemed exempt from federal human subjects research regulations by the Georgia State University Institutional Review Board.

### 2.2 Measures

Sociodemographic variables included age, race/ethnicity, education, annual household income, employment status, health insurance status, gender, and sexual orientation (Table 1). An adapted Domain-Specific Stress Scale measured COVID-19-related worries. (University of Southern California Center for Population Health, 2021) Participants rated their agreement (−2 = Strongly disagree to 2 = Strongly agree) with: “I am worried about…” (a)“not having enough to eat or to feed my family;” (b) “being evicted or losing my home, or not having a place to live;” (c) “losing my job or having my hours and pay reduced;” (d) “not having enough money to live;” (e) “going to doctors’ appointments or to the pharmacy to pick up my prescriptions because of coronavirus;” (f) “affording medical care;” (g)“I am feeling very isolated from family/friends or feel very lonely;” and (h) “I am worried about protests or experiences with racism, discrimination, or police injustice.” Responses were dichotomized to indicate agreement with each worry (“agree” and “strongly agree” vs. all other answers).

**Table 1:** **COVID-19-Related Worries and Distress by Sociodemographic Characteristics among U.S. Adult Smokers, 2020,** N = 1,223.

	**Food Insecurity**	**Housing** **Instability**	**Job** **Insecurity**	**Financial Concerns**	**Difficulty Accessing Healthcare**	**Medical Care Not Affordable**	**Isolation/ Loneliness**	**Racism, Discrimination, Police Injustice**	**Serious Psychological Distress**
	Weighted % (95% CI) or *p*	Weighted % (95% CI) or *p*	Weighted % (95% CI) or *p*	Weighted % (95% CI) or *p*	Weighted % (95% CI) or *p*	Weighted % (95% CI) or *p*	Weighted % (95% CI) or *p*	Weighted % (95% CI) or *p*	Weighted % (95% CI) or *p*
**Age**	<0.001	<0.001	<0.001	0.19	0.17	0.21	0.16	0.20	<0.001
18–29	14.71 (7.21, 22.2)	10.54 (3.97, 17.11)	33.79 (23.37, 44.21)	38.83 (28.02, 49.64)	19.01 (10.38, 27.64)	34.49 (23.81, 45.17)	41.75 (30.91, 52.59)	37.25 (26.48, 48.03)	20.85 (11.85, 29.86)
30–44	28.67 (22.7, 34.64)	21.21 (15.71, 26.7)	26.1 (20.39, 31.8)	42.43 (36.11, 48.75)	29.69 (23.63, 35.75)	35.6 (29.45, 41.76)	32.69 (26.66, 38.72)	38.19 (31.95, 44.43)	23.74 (17.82, 29.67)
45–59	23.6 (19.02, 28.18)	17.68 (13.58, 21.78)	23.3 (18.83, 27.76)	42.16 (36.8, 47.52)	26.17 (21.44, 30.89)	37.87 (32.6, 43.13)	32.24 (27.26, 37.23)	45.2 (39.82, 50.58)	12.41 (8.97, 15.86)
60+	12.05 (8.64, 15.46)	8.66 (6.07, 11.24)	13.97 (10.25, 17.7)	30.78 (25.91, 35.66)	24.43 (19.95, 28.91)	25.59 (21.08, 30.09)	30.33 (25.75, 34.92)	47.05 (41.84, 52.26)	5.1 (3.08, 7.13)
**Race/ethnicity**	0.03	0.06	0.04	0.33	0.08	0.06	0.25	0.72	0.77
White, NH	19.31 (16.21, 22.42)	13.84 (11.11, 16.58)	21.75 (18.37, 25.14)	37.66 (33.73, 41.59)	22.93 (19.58, 26.28)	34.45 (30.56, 38.35)	34.99 (31.13, 38.84)	40.24 (36.35, 44.13)	15.52 (12.32, 18.72)
Black, NH	21.45 (14.49, 28.41)	15.49 (9.6, 21.37)	22.76 (14.5, 31.02)	42.03 (32.44, 51.62)	32.3 (23.57, 41.03)	28.63 (20.13, 37.13)	30.41 (21.42, 39.4)	44.42 (34.7, 54.15)	13.75 (5.95, 21.56)
Other, NH	17.46 (4.27, 30.65)	12.59 (1.3, 23.88)	29.16 (13.73, 44.6)	37.08 (21.84, 52.31)	28.42 (14.19, 42.64)	22.54 (9.07, 36.02)	25.66 (12.64, 38.68)	49.06 (33.18, 64.95)	20.87 (7.46, 34.28)
Hispanic/ Latinx	31.47 (20.12, 42.81)	26.16 (15.47, 36.84)	38.03 (26.38, 49.68)	45.79 (34.18, 57.4)	30.96 (19.81, 42.1)	43.02 (31.39, 54.66)	35.91 (24.32, 47.5)	45.21 (33.65, 56.78)	17.39 (8.02, 26.75)
**Education**	<0.001	<0.001	0.02	<0.001	<0.01	<0.001	0.66	0.09	<0.001
< High School	33.66 (23.88, 43.45)	23.72 (14.73, 32.7)	24.18 (14.86, 33.49)	55.18 (45.04, 65.32)	29.4 (19.82, 38.99)	38.82 (28.72, 48.92)	30.62 (20.93, 40.31)	35.82 (25.94, 45.69)	27.37 (17.47, 37.28)
High School	18.04 (13.82, 22.26)	12.72 (9.21, 16.23)	21.5 (16.31, 26.69)	37.52 (31.85, 43.19)	24.62 (19.76, 29.48)	36.17 (30.41, 41.93)	34.5 (28.86, 40.14)	41.6 (35.81, 47.4)	13.99 (9.63, 18.36)
Some College	21.14 (16.92, 25.36)	16.47 (12.52, 20.42)	28.65 (23.67, 33.64)	39.11 (33.88, 44.35)	23.7 (19.26, 28.14)	33.58 (28.6, 38.56)	33.81 (28.75, 38.86)	43.72 (38.42, 49.02)	13.25 (9.65, 16.85)
Bachelor’s degree or higher	10.38 (5.28, 15.48)	8.44 (4.1, 12.77)	20 (13.52, 26.48)	21.38 (14.78, 27.98)	27.01 (19.03, 34.99)	20.45 (14.09, 26.81)	36.53 (28.27, 44.79)	46.53 (37.93, 55.12)	12.85 (6.61, 19.09)
**Household Income**	<0.001	<0.001	<0.001	<0.001	<0.001	<0.001	<0.001	0.03	<0.001
< $30,000	31.96 (26.13, 37.8)	23.11 (17.78, 28.44)	26.36 (20.53, 32.2)	57.04 (50.69, 63.39)	32.02 (26.16, 37.87)	37.12 (30.88, 43.37)	39.27 (32.9, 45.63)	39.44 (33.27, 45.61)	24.00 (18.32, 29.69)
$30,000 - $99,999	19.76 (15.76, 23.75)	13.71 (10.4, 17.01)	24.17 (19.75, 28.59)	35.70 (31.03, 40.36)	22.08 (18.11, 26.05)	35.71 (31.1, 40.32)	32.17 (27.73, 36.61)	42.84 (38.07, 47.6)	13.29 (9.6, 16.97)
≥ $100,000	9.68 (4.77, 14.59)	9.31 (4.36, 14.27)	21.63 (15.07, 28.2)	24.52 (17.39, 31.65)	24.13 (17.2, 31.06)	26.27 (18.78, 33.77)	30.78 (23.4, 38.15)	43.12 (35.36, 50.88)	11.27 (5.27, 17.26)
**Employment**	<0.001	<0.001	<0.001	<0.001	<0.001	<0.001	0.04	0.03	0.01
Working	19.35 (15.84, 22.86)	15.39 (12.3, 18.48)	30.84 (26.68, 35)	33.85 (29.63, 38.07)	23.64 (19.78, 27.5)	35.14 (30.82, 39.45)	32.14 (27.98, 36.31)	41.32 (36.92, 45.72)	14.48 (10.97, 17.98)
Not working- layoff/looking for work	24.76 (13.07, 36.45)	21.91 (10.41, 33.41)	27.01 (14.16, 39.86)	56.87 (42.74, 71)	16.65 (6.71, 26.59)	32.87 (19.25, 46.48)	39.27 (25.04, 53.49)	42.46 (28.32, 56.61)	22.27 (9.7, 34.84)
Not working-retired	14.09 (9.43, 18.74)	9.27 (5.67, 12.88)	5.66 (1.95, 9.37)	34.78 (28.09, 41.46)	27.51 (21.62, 33.39)	26.72 (20.75, 32.69)	30.31 (24.51, 36.1)	44.69 (38.04, 51.34)	8.62 (5.21, 12.03)
Not working- disabled	33.11 (23.46, 42.76)	16.23 (8.39, 24.07)	7.53 (2.27, 12.78)	60.81 (50.55, 71.08)	39.21 (28.83, 49.6)	34.14 (24.05, 44.22)	44.23 (33.68, 54.77)	41.11 (30.94, 51.27)	28.36 (18.80, 37.92)
Not working-other	29.8 (11.87, 47.73)	18.88 (2.05, 35.71)	20.75 (2.55, 38.96)	44.69 (26.64, 62.75)	33.19 (15.32, 51.05)	40.47 (22.42, 58.52)	36.94 (18.57, 55.3)	42.07 (23.96, 60.19)	20.13 (3.62, 36.65)
**Health Insurance**	<0.001	<0.001	<0.001	<0.001	<0.001	<0.001	<0.001	<0.001	<0.001
Private or insured	12.91 (9.64, 16.18)	11.65 (8.53, 14.77)	25.38 (20.76, 30.00)	30.05 (25.24, 34.87)	20.26 (16.19, 24.33)	33.13 (28.18, 38.09)	30.75 (26.05, 35.45)	41.78 (36.63, 46.93)	10.15 (6.72, 13.57)
Medicare	21.32 (14.74, 27.89)	12.78 (7.36, 18.2)	12.11 (6.31, 17.91)	42.86 (35.8, 49.93)	36.63 (29.57, 43.7)	32.63 (25.73, 39.52)	34.14 (27.36, 40.92)	50.29 (43.34, 57.24)	15.55 (9.12, 21.99)
Medicaid	37.81 (29.83, 45.79)	22.77 (15.96, 29.58)	26.74 (19.52, 33.96)	59.25 (51.16, 67.34)	34.33 (26.38, 42.28)	31.11 (23.22, 39.01)	46.97 (38.65, 55.29)	40.35 (32.36, 48.33)	29.64 (21.69, 37.6)
VA/DOD/ Military Program	8.54 (1.83, 15.24)	8.54 (1.83, 15.24)	13.98 (4.33, 23.64)	18.31 (7.51, 29.1)	12.83 (3.55, 22.12)	10.9 (2.44, 19.35)	28.91 (14.65, 43.18)	34.8 (19.87, 49.73)	23.65 (4.38, 42.92)
Uninsured	28.91 (19.28, 38.53)	23.74 (14.55, 32.94)	32.02 (21.64, 42.4)	44.64 (34.07, 55.21)	22.89 (13.73, 32.05)	49.15 (38.54, 59.75)	28.86 (18.41, 39.32)	38.27 (27.74, 48.81)	17.51 (9.19, 25.82)
**Gender**	0.03	0.45	0.43	0.03	0.03	0.10	0.01	0.18	<0.001
Male	17.37 (13.75, 21.00)	13.91 (10.7, 17.12)	24.1 (20.93, 27.27)	35.79 (31.08, 40.5)	21.58 (17.59, 25.56)	33.36 (28.64, 38.07)	29.32 (24.88, 33.77)	39.12 (34.35, 43.89)	11.35 (7.90, 14.8)
Female	24.88 (20.5, 29.25)	17.01 (13.09, 20.92)	29.99 (11.94, 48.04)	43 (38.04, 47.96)	29.97 (25.39, 34.55)	34.39 (29.67, 39.11)	39.25 (34.33, 44.16)	45.28 (40.35, 50.21)	21.22 (16.75, 25.7)
**Sexual Orientation**	0.22	0.67	0.47	0.43	0.87	0.21	0.12	0.08	0.28
Straight, that is, not gay	20.43 (17.59, 23.27)	15.43 (12.9, 17.95)	24.1 (20.93, 27.27)	38.79 (35.3, 42.27)	25.48 (22.41, 28.56)	34.05 (30.63, 37.46)	33.65 (30.28, 37.03)	42.15 (38.65, 45.64)	15.38 (12.58, 18.18)
Not Straight	32.59 (14.29, 50.9)	15.61 (0, 32.55)	29.99 (11.94, 48.04)	44.72 (25.92, 63.51)	24.29 (6.86, 41.71)	31.18 (13.19, 49.17)	37.84 (19.45, 56.23)	39.64 (21.4, 57.88)	24.15 (5.66, 42.63)

*Note.* CI = confidence interval, VA = Veteran Affairs, DOD = Department of Defense. P values indicate significant associations between sociodemographic characteristics and agreement with experiencing each of the COVID-19-related worries, as determined by Rao-Scott Chi-Square. Serious psychological distress is indicated by scores of 13 or higher on the Kessler-6 (K-6) scale. Percent represented is those who agree.

The Kessler-6 scale assessed past-month psychological distress. Items were summed, with scores greater than or equal to 13 indicating serious psychological distress (SPD). (Kessler et al., 2003).

### 2.3 Data analyses

Rao-Scott Chi-square analyses examined associations between sociodemographic variables with each COVID-19 worry and SPD. Weighted logistic regression analyses predicted SPD from COVID-19 worries. Analyses were conducted with SAS 9.4.

## 3 Results

The sample included 1,223 current smokers (53% male, mean age 52.64 [SD = 14.52]). Approximately 69% identified as non-Hispanic white, 13% non-Hispanic Black, 11% Hispanic, and 7% “other” race/ethnicity. Forty-four percent had attained less than a high school education; 47% reported annual household income between $30,000-$99,999; 61% were currently employed; and 46% had private health insurance.

Participants aged 30–44 reported more worry about food and housing, and those aged 18–29 had greater worry about jobs (vs. ages 60+, Table 1). More Hispanic/Latinx participants reported worries about jobs than non-Hispanic whites. Worries about food, housing, finances, and affording medical care were more prevalent among those with less than a high school education (vs. bachelor’s degree or greater). Worry in most domains was more widespread among those with household income less than $30,000 and unemployed (laid off/looking for work or disabled). Compared to those with private insurance, participants on Medicaid were more likely to report worry about food insecurity, finances, and isolation/loneliness. Women were more likely than men to feel isolated or lonely. There were no associations between sexual orientation and any of the worries. Race/ethnicity was associated with worries about food insecurity and job insecurity, but not other worries.

Women, participants aged 30–44, those with income less than $30,000, less than high school education, not working/disabled, or on Medicaid were more likely to be experiencing SPD. Each COVID-19-related worry predicted higher likelihood of SPD (*p*s < 0.05), even after controlling for sociodemographics. When all COVID-19 worries were entered simultaneously, worries about finances (aOR = 2.30, 95% CI 1.23–4.29, *p* <.001) and isolation/loneliness (aOR = 3.04, 95% CI 1.80–5.16, *p* <.001) uniquely predicted SPD.

## 4 Discussion

Among this nationally representative sample of U.S. adult smokers, those with lower SES disproportionately experienced worry about access to basic needs (e.g., food, housing, healthcare) and were more likely to be experiencing SPD during the COVID-19 pandemic. This includes those with lower income and education; those not working; and those on Medicaid. A higher proportion of Hispanic/Latinx smokers reported worries regarding job insecurity. The pandemic may be magnifying health disparities for these subgroups. Participants under age 44 were more likely to worry about food, housing, and jobs, which may be because they are in critical periods of building careers and families.

Our findings have important implications for health disparities during the pandemic. Low-SES smokers experience disproportionately high stress and low healthcare access, both of which impeded smoking cessation before COVID-19. (Hiscock et al., 2012) Low-SES smokers are experiencing even greater stress and difficulty accessing medical care now. Although the vulnerability of smokers to COVID-19 could be motivating for some to quit smoking, stress is a common reason for increased smoking during the pandemic. (Yingst et al., 2021) The association between COVID-19 worries and SPD should raise concern about poor mental health and poor smoking cessation outcomes.(Streck et al., 2020) Efforts are needed to address pandemic-related stress in low-SES smokers. This could include policies that increase access to basic needs, mental healthcare and smoking cessation services; community and organizational efforts to address stress and isolation; and individual stress management interventions.

Surprisingly, race/ethnicity was not associated with worry about racism, discrimination, and police injustice. Between 40 and 49% of participants (regardless of race/ethnicity) indicated current worry about these issues. Recent protests and heightened awareness of racism may have led to relatively high stress about these issues across racial and ethnic categories. Notably, the survey item did not specify if worries about racism were related to self or others. Future research could include more specific items about experiences, worries, and attributions for different types of discrimination.

This study is limited by its cross-sectional design and reliance on self-report. We surveyed participants’ reports of worry about basic needs rather than objective assessments of hardship. Moreover, because the survey only asked about stress and worry since February 2020, we are not able to compare these stress levels to those before the pandemic. However, this study is strengthened by a large and timely nationally representative survey of adult smokers, who are at particular risk for COVID-19-related complications. The findings help identify how COVID-19 has impacted historically and systemically marginalized communities, and the types of stressors that these populations are experiencing during the pandemic. Given the association of COVID-19 worries with SPD, the need for mental health resources among marginalized groups is essential.

## 5 Public health implications

Among U.S. adult smokers during the COVID-19 pandemic, worry about access to basic needs (e.g., food, housing, healthcare) and SPD are particularly prevalent among those with lower SES. Policies and interventions that address basic needs and mental health among marginalized populations of tobacco users are needed.

## 6 Funding statement

This research was supported by the Robert Wood Johnson Foundation. The content is solely the responsibility of the authors and does not necessarily represent the official views of the Robert Wood Johnson Foundation.

## Declaration of Competing Interest

The authors declare that they have no known competing financial interests or personal relationships that could have appeared to influence the work reported in this paper.
